# Adipokines Do Not Mediate the Association of Obesity and Colorectal Adenoma

**DOI:** 10.1155/2014/371254

**Published:** 2014-08-13

**Authors:** Heather M. Ochs-Balcom, Rikki Cannioto, Jing Nie, Amy E. Millen, Jo L. Freudenheim, Zhengyi Chen, Cheryl L. Thompson, Russell Tracy, Li Li

**Affiliations:** ^1^Department of Epidemiology and Environmental Health, School of Public Health and Health Professions, University at Buffalo, Buffalo, NY 14214, USA; ^2^Department of Family Medicine and Community Health, Case Western Reserve University, Cleveland, OH 44106, USA; ^3^Department of Biochemistry, University of Vermont, Burlington, VT 05405, USA

## Abstract

*Purpose*. The association between obesity and colon neoplasia is well established but the underlying biological mechanisms are not fully understood. Rates of both obesity and colon cancer differ by race. Adipokines have been postulated as contributors to the observed association; however, few studies have examined the mediating effect of adipokines on the obesity-colon adenoma association with consideration of racial differences. *Methods*. We determined prediagnostic levels of adiponectin and leptin in Caucasians (217 cases and 650 controls) and African Americans (175 cases and 378 controls) participating in the *Case Transdisciplinary Research on Energetics and Cancer Colon Adenoma Study*. We evaluated mediating effects of adiponectin and leptin on the association of abdominal adiposity and colon adenoma separately according to race using mediational pathway analysis. *Results*. We observed differences in circulating adipokine concentrations by race; African Americans had higher levels of leptin and lower levels of adiponectin than Caucasians for both adenoma cases and controls (*P* values <0.001). Leptin and adiponectin did not mediate the waist-to-hip ratio (WHR) adenoma association in either group (all Sobel *P* values >0.27). *Conclusions*. We found no evidence that leptin or adiponectin mediates the abdominal obesity-colorectal adenoma pathway. Larger studies on how these associations vary by race, sex, and obesity are needed.

## 1. Introduction

Colorectal cancer is the third most common cancer in men and women and the second leading cause of cancer deaths in the United States (US) [1]. Racial disparities in incidence and mortality are well recognized; African Americans disproportionately experience the highest colorectal incidence and mortality rates compared to all other race/ethnic groups in the US [1–3]. It is well recognized that reasons for such disparities are complex and multifactorial. Socioeconomic factors, differential stage at diagnosis, and access to medical care are important factors, but they do not fully account for observed disparities [4, 5]. Other factors such as nutrition, physical activity, obesity, and cultural factors that influence medical decision making before and after diagnosis are currently under study [6].

Obesity, abdominal obesity in particular, is particularly appealing for study as a contributor to cancer health disparities as a potentially modifiable risk factor. While obesity is positively associated with both colon adenomas and colon cancer [7–10], there is a paucity of published data regarding differential associations of obesity and adenoma polyps with race. We previously showed that waist-to-hip ratio (WHR) in particular is positively associated with colon adenomas for both European and African Americans [11]. Biologic mechanisms for the associations of abdominal adiposity and colon adenoma are not completely understood; mechanisms including inflammation and insulin resistance are currently under study [12, 13]. An additional possible mechanism is mediation by leptin and adiponectin. Further, because of known differences in these adipokines by race [14], examination of these relationships within race groups is important.

Herein we extend our previous work on the association of obesity and adenoma [11, 15] by analyzing whether adiponectin and leptin mediate the relationship between adiposity and colon adenoma in a sample of Caucasians and African Americans.

## 2. Methods

### 2.1. Study Population

Participants were recruited to the* Case Transdisciplinary Research on Energetics and Cancer (TREC) Colon Polyps Study*, an ongoing screening colonoscopy-based study as previously described [15]. In brief, potential participants scheduled for routine colonoscopy at University Hospitals Case Medical Center and/or affiliated endoscopy centers were initially screened by telephone to determine eligibility. We excluded individuals younger than 30 years of age and those reporting a personal history of any cancer, ulcerative colitis, Crohn's disease, or previous colorectal adenoma, as well as individuals who reported a family history of hereditary nonpolyposis colon cancer or familial adenomatous polyposis. This study was approved by the University Hospitals Case Medical Center and University at Buffalo Institutional Review Boards and all participants provided written informed consent.

Data on demographic characteristics and colon cancer risk factors were collected before the colonoscopy via a phone survey and were based on the lifestyle risk factor questionnaire developed by the colon cancer family registry group (http://www.coloncfr.org/index.php/questionnaires). Participants also completed three additional questionnaires which were developed and validated by the Arizona Diet, Behavior, and Quality of Life Assessment Center [16]: the Arizona Food Frequency, Physical Activity, and Meat Preparation questionnaires. Data on smoking history and NSAID use (ever taken aspirin/ibuprofen at least twice a week for >1 month) were also collected as part of the phone survey. On the day of the colonoscopy, a research nurse obtained a fasting blood sample for measurement of leptin and adiponectin and performed anthropometric measurements. Anthropometric data allowed for calculation of body mass index (BMI, kg/m^2^) and waist-to-hip ratio (WHR).

Our study sample included 1527 Caucasians and African Americans in total, including 421 incident colorectal adenoma cases and 1106 adenoma-free controls. We excluded 95 (6.2%) participants who were missing leptin and adiponectin measurements and 12 (1.0%) who were missing anthropometric measurements, resulting in a final sample size of 217 Caucasian polyp cases, 650 Caucasian controls, 175 African American polyp cases, and 378 African American controls.

### 2.2. Laboratory Methods

For measurement of leptin and adiponectin, EDTA plasma samples were assayed with solid phase quantitative sandwich ELISA (R&D systems, Minneapolis, MN) according to standard protocol. Frozen pools and lyophilized control materials were used in each assay. All assays were carried out blinded to case-control status. Leptin and adiponectin interassay coefficients of variation ranged from 4.9 to 8.1% and from 10.0 to 11.3%, respectively.

### 2.3. Statistical Analysis

Descriptive statistics, stratified by case-control status and race, were estimated using *t*-tests for continuous variables and chi-square tests for categorical variables; the *P* value for statistical significance was determined* a priori* at less than 0.05. We developed general linear models to evaluate linear trends in waist-to-hip ratio (WHR) across quartiles of leptin and adiponectin, using quartile cutoffs estimated from the control group with adjustment for age and sex.

We evaluated whether adiponectin and leptin attenuate the association of WHR and adenoma in logistic models stratified by race. We also applied formal Sobel mediation tests using three steps [17]: (1) evaluate the degree to which each adipokine is independently associated with WHR by linear regression with leptin or adiponectin as the dependent variable and WHR as the independent variable, (2) evaluate the degree to which the adipokine is associated with adenoma by logistic regression with adenoma as the dependent variable and leptin or adiponectin as the independent variable (including WHR), and (3) use the estimated regression coefficients and their standard errors from Steps 1 and 2 to calculate the critical ratio and test whether the indirect effect of WHR on adenoma via leptin or adiponectin is significantly different from zero. We used IBM SPSS Version 21 for all analyses [18].

## 3. Results

We observed several differences by race among incident adenoma cases. African American adenoma cases had a higher BMI (31.7 versus 29.1 kg/m^2^), higher frequency of individuals with BMI > 30 kg/m^2^ (51% versus 36%), and a higher mean WHR and waist circumference compared to Caucasian adenoma cases (Table 1). With regard to adiponectin and leptin, we observed lower mean values of adiponectin and higher values of leptin and fasting glucose in African American cases compared to Caucasian cases. African American cases were less likely to report a family history of colorectal cancer (17% versus 27% for Caucasians) and more likely to be current smokers (35% versus 13% for Caucasians). Among adenoma-free controls, there were similar differences in measures of obesity, adiponectin and leptin, fasting glucose, insulin, and smoking status between African Americans and Caucasians.

While African American cases were slightly older than African American controls (*P* = 0.001), African American cases and controls were not different in BMI but cases averaged higher WHR compared to controls (0.97 versus 0.93, *P* = 0.001). Average leptin values were lower (*P* = 0.006) and adiponectin levels were not statistically significantly different (*P* = 0.36) in African American cases compared to African American controls.

Differences between Caucasian cases and controls included age (cases were slightly older than controls, *P* = 0.001), BMI (29.1 versus 28.0 kg/m^2^, for cases and controls, resp., *P* = 0.003), and WHR and waist circumference (both *P* = 0.001). We observed no statistically significant differences in mean leptin (*P* = 0.92) or adiponectin (*P* = 0.07) between Caucasian cases and controls.

**Figure 1:**
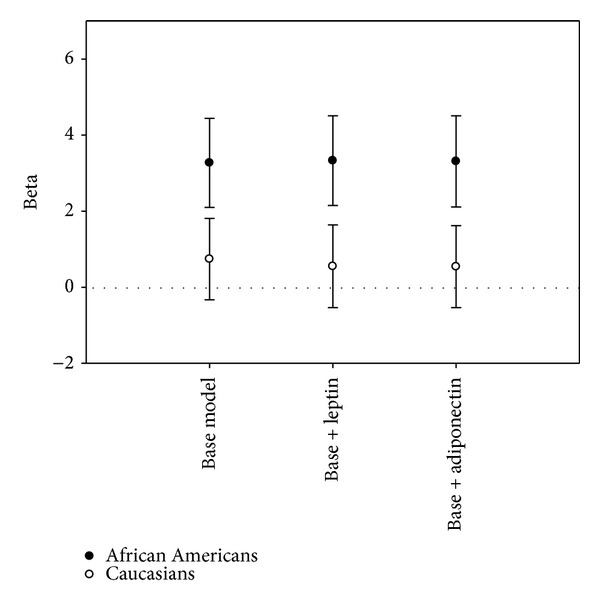
Beta coefficients (95% confidence intervals) for the association between WHR and adenoma stratified on race and adjusted for age and sex, examining possible mediation by leptin and adiponectin. WHR, leptin, and adiponectin entered as continuous variables.

The association of  WHR and adenoma for African Americans and Caucasians, adjusted for age and sex, is shown in Figure 1. These analyses include the hypothesized mediation of leptin and adiponectin. Across both groups it can be seen that adding leptin or adiponectin to the models has negligible or very minimal attenuation on the association of WHR and polyps.

Step 1 of the formal mediation analysis was supportive of an association between WHR and adiponectin for both African Americans and Caucasians, whereas WHR was associated with leptin in Caucasians only (Table 2). In the second step of the mediation analysis, we tested the association of each adipokine with adenoma and we found no statistically significant associations. Using the estimates from Steps 1 and 2 to formally test for mediation, we found no evidence for statistical significance for a mediating effect of adipokine in either African Americans or Caucasians.

## 4. Discussion

We observed several interesting and statistically significant racial differences in adiponectin, as well as associations between abdominal obesity and both adipokines in the direction we expected. The Sobel mediation analysis relies on estimates of the association between obesity (WHR) and adipokines and adenoma status. In our study we observed no associations of either leptin or adiponectin with adenoma status for either Caucasians or African Americans; therefore our study does not support a mediating effect of either leptin or adiponectin in the obesity-adenoma pathway.

The association between circulating leptin and colorectal adenomas has been investigated in three epidemiologic studies [19–21]. Chia et al., 2007, found increased adenoma risk for men but not women with the highest leptin (OR = 3.3, 95% CI: 1.2–8.7; *P* trend = 0.01); the association was attenuated to OR = 2.3, 95% CI: 0.7–7.7, and was no longer significant with BMI adjustment [19]. This study included primarily Caucasians. The second study, a case-control study of Japanese men and women, reported no association between leptin and adenoma risk when cases and controls were age-, sex-, and BMI-matched [20]. More recently, a case-control study of Japanese men and women reported a statistically significant association between adenoma risk and circulating leptin levels among men, but not women [21]. After adding BMI to the model, ORs were attenuated among men but remained borderline statistically significant (OR = 1.44, 95%: 0.99–2.08). These studies are consistent in that adjustment for BMI mitigates an association of leptin and adenoma.

A recent meta-analysis was conducted to analyze the association of circulating leptin and both colorectal adenoma and colorectal cancer [22]. While the individual studies did not show higher relative risk for increasing leptin concentration, the meta-analysis suggested a 35% increased adenoma risk for the highest versus the lowest leptin categories (RR: 1.35, 95% CI: 1.03–1.76) and no association of leptin and colorectal cancer. Additional and larger studies of leptin and adenoma are needed to obtain more representative and stable estimates and to examine differences within subgroups including sex and race.

Three of the four studies assessing circulating adiponectin and adenoma risk yielded statistically significant inverse associations. In each of three case-control studies of Japanese men and women there was a statistically significant association between circulating adiponectin and adenoma risk, with decreases in odds of adenoma ranging between 44% and 76% with every 1 *μ*g/mL increase in total adiponectin [20, 21, 23]. The one prospective study, which included male participants only, did not report statistically significant associations [24]. Furthermore, two studies assessed the relationship between high molecular weight adiponectin and adenomas, but neither study observed statistically significant associations [21, 24].

Two meta-analyses of adiponectin concentrations and colorectal cancer and adenomas were recently published and analyzed similar published literature [25, 26]. The first meta-analysis included 13 studies totaling 6,175 participants (3015 colorectal cancer cases and/or adenoma cases) [26]. The weighted mean differences between cases and controls were −1.084 *μ*g/mL (95% CI: −1.84–0.33, *P* = 0.05) for colorectal cancer cases and −1.43 *μ*g/mL (95% CI: −2.23–0.63, *P* = 0.0001) for adenoma cases [26]. Sex-stratified analyses yielded a 2% decreased risk of colorectal neoplasm for a 1 *μ*g/mL increase in adiponectin (OR = 0.98, 95% CI: 0.96–0.99) among men, but a statistically significant trend was not observed for women (OR = 0.99, 95% CI: 0.97–1.01) [26]. These authors concluded that colorectal cancer and adenoma cases have “markedly lower” adiponectin concentrations and, more specifically, that a negative dose-response relationship was evident in men. A second meta-analysis included 2632 cases of colorectal cancer or adenoma and 2753 healthy controls [25]; findings of this meta-analysis suggest that adiponectin levels are statistically significantly lower in patients with colorectal cancer or adenoma compared with controls. A weighted mean difference of −1.51 (95% CI: −2.42–0.59), *P*_heterogeneity_ < 0.0001, was observed in colorectal cancer in comparison to controls, and a weighted mean difference of −1.29 (95% CI: −2.01–0.58), *P*_heterogeneity_ < 0.0001, was observed in adenoma cases [25]. The authors cautioned that case-control studies with smaller sample sizes yielded the greatest mean differences and that additional larger-scale epidemiological studies were warranted [25].

To date, one study assessed the role of adipokines as potential mediating factors in the obesity-colorectal cancer pathway [27]. In this study of postmenopausal women, Ho et al. found that leptin, and not adiponectin, mediated the association between abdominal obesity and colorectal cancer risk. Their primary outcome of interest was colorectal cancer, whereas our outcome was adenoma. Their sample was comprised of primarily white (postmenopausal) women while our study represents adult Caucasian and African American men and women. The current literature and our own data suggest that postmenopausal women have higher leptin concentrations. The obesity-colorectal cancer association may be more strongly influenced by adipocyte dysfunction and adipokine dysregulation, and the mechanism for the obesity-precancerous adenoma pathway, initiated as much as 10 years earlier, may be different.

Much of the epidemiological data examining the obesity-adipokine-colorectal cancer pathway has been conducted with Asian populations, and very few studies have been published using US samples where obesity rates may be higher. To our knowledge, ours is the first US-based epidemiological study including a large sample of African Americans; it is possible that differences in findings may be reflective of both environmental and racial differences related to obesity which have not yet been fully elucidated.

Epidemiological evidence suggests that circulating plasma concentrations of adiponectin and leptin may not precisely reflect an individual's true or long-term levels [14, 27]. Also, for certain adipokines, the adipokine tissue concentrations in the tumor environment may be more relevant to the assessment of cancer related risk than circulating blood levels. Recent epidemiological evidence has suggested only a moderate correlation between plasma and breast tissue leptin and a weak correlation between plasma adiponectin and breast tissue adiponectin [14]; it is not known if there is a similar lack of correlation between blood and colon tissue concentrations. This discordance between plasma and tissue concentrations of adiponectin and leptin may, in part, explain why some studies have not observed associations between blood concentrations of adipokines and colorectal cancer risk. Finally, relying on total adiponectin, as opposed to identifying more bioactive components of adiponectin, may also explain some of the inconsistencies in the literature and our null findings.

Our study provides new important data because it includes a large sample of both African American and Caucasian participants. To our knowledge, this is the largest and most diverse US epidemiologic study of the relationship between obesity, adiponectin, leptin, and adenoma. Additional strengths of the study include the direct measurement of anthropometric variables by study nurses and the inclusion of data representing all well-established risk and protective factors associated with colorectal cancer (i.e., smoking, NSAID use, alcohol, physical activity, family history, and several measures of adiposity).

Weaknesses of the study include the reliance on a single measure of each of the adipokines taken from blood samples collected at the time of colonoscopy. Furthermore, reliance upon total adiponectin (as opposed to high molecular versus low molecular weight adiponectin concentrations) could be a limitation given recent evidence suggesting that the high molecular weight and low molecular weight components of adiponectin may have different biological functions. Lastly, reduced sample sizes for subset analyses may have limited our power to detect statistically significant associations.

## 5. Conclusions

Our study is in agreement with previous evidence that circulating blood concentrations of adiponectin and leptin vary by race and obesity. We did not find evidence of a mediating effect of leptin or adiponectin in the obesity-adenoma association. A better understanding of the association between obesity, adiponectin, and leptin and adenoma and how these associations vary by race, sex, and obesity is needed to further understand the role that adipokines, recognized as important biologic components of the obesity phenotype, may play in association with cancer risk.

## Figures and Tables

**Table 1:** Descriptive characteristics for TREC adenoma study.

Characteristics Mean (SD) or *n* (%)	African American adenoma cases (*n* = 175)	Caucasian adenoma cases (*n* = 217)	*P* value	African American adenoma-free controls (*n* = 378)	Caucasian adenoma-free controls (*n* = 650)	*P* value
Age (years)	58.2 (8.4)	57.4 (8.1)	0.35	54.7 (8.6)	54.4 (8.6)	0.50
Male	71 (40.6%)	113 (52.1%)	0.02	91 (24.1%)	240 (36.9%)	<0.001
Body mass index (BMI) (kg/m^2^)	31.7 (7.3)	29.1 (5.5)	<0.001	32.3 (7.4)	28.0 (5.7)	<0.001
BMI categories						
≤24.9	26 (14.9%)	45 (20.7%)	0.01	45 (11.9%)	207 (31.9%)	<0.001
25.0–29.9	59 (33.7%)	94 (43.3%)		122 (32.3%)	243 (37.4%)	
30.0–34.9	45 (25.7%)	48 (22.1%)		91 (24.1%)	126 (19.4%)	
35.0–39.9	24 (13.7%)	20 (9.2%)		71 (18.8%)	44 (6.8%)	
≥40.0	21 (12.0%)	10 (4.6%)		49 (13.0%)	30 (4.6%)	
Waist-to-hip ratio (WHR)	0.97 (0.09)	0.92 (0.09)	<0.001	0.93 (0.09)	0.90 (0.09)	<0.001
Waist circumference (cm)	105.0 (17.1)	99.6 (16.0)	0.001	104.1 (16.7)	95.1 (16.1)	<0.001
Abdominal obesity^1^	125 (71.4%)	124 (57.1%)	0.003	279 (73.8%)	318 (48.9%)	<0.001
Adiponectin (ng/mL)	7757.0 (5437.4)	10577.1 (5732.9)	<0.001	8017.6 (5166.1)	11637.9 (6443.4)	<0.001
Leptin (pg/mL)	25341.3 (23384.2)	16012.3 (17593.0)	<0.001	31458.6 (25700.5)	16158.4 (16707.8)	<0.001
Fasting glucose (mg/dL)	99.9 (29.8)	86.2 (17.0)	<0.001	94.0 (36.8)	85.1 (24.0)	<0.001
Insulin (*µ*IU/mL)	15.3 (61.2)	7.7 (11.7)	0.11	9.9 (14.1)	6.2 (7.1)	<0.001
Total daily energy expenditure (kj)	10485.2 (3005.5)	10615.1 (2829.2)	0.68	10574.0 (3404.7)	10627.7 (2877.8)	0.81
Diabetes^2^	44 (25.2%)	17 (7.8%)	<0.001	85 (22.6%)	56 (8.6%)	<0.001
Postmenopausal	81 (46.8%)	73 (33.6%)	0.03	204 (54.3%)	254 (39.1%)	<0.001
NSAID use^3^	66 (37.7%)	88 (40.6%)	0.57	133 (35.2%)	228 (35.1%)	0.97
Family history of colorectal cancer	29 (16.9%)	58 (27.0%)	0.02	90 (24.4%)	160 (24.8%)	0.87
Pack-years of smoking	20.8 (18.7)	20.3 (19.9)	0.83	14.9 (14.6)	17.2 (22.1)	0.15
Smoking status						
Never	52 (29.7%)	97 (44.7%)	<0.001	147 (39.0%)	332 (51.1%)	<0.001
Former	61 (34.9%)	91 (41.9%)		132 (35.0%)	257 (39.5%)	
Current	62 (35.4%)	29 (13.4%)		98 (26.0%)	61 (9.4%)	

^1^Abdominal obesity defined as waist circumference >88 cm for women and >102 cm for men.

^2^Diabetes was self-reported.

^3^NSAID use defined as ever taken aspirin/ibuprofen at least twice per week for >1 month.

**Table 2:** Race-stratified mediation analysis [17].

Race	Biomarker	Step 1 *β* (SE)^1^	Step 1 *P* value	Step 2 *β*′ (SE)^2^	Step 2 *P* value	Step 3 Sobel mediation *P* value
African Americans	Leptin	8313.5 (11456.5)	0.47	−0.000005 (0.000005)	0.31	0.56
	Adiponectin	−13743.7 (2515.4)	<0.001	0.000004 (0.000019)	0.84	0.83

Caucasians	Leptin	39913.3 (7003.6)	<0.001	0.000005 (0.000005)	0.36	0.32
	Adiponectin	−14566.5 (2497.2)	<0.001	−0.000016 (0.000015)	0.30	0.29

^1^Linear regression of WHR (independent variable) and adipokine (dependent variable) adjusted for age and sex.

^2^Logistic regression of adipokine (independent variable) and adenoma (dependent variable) adjusted for age, sex, and WHR.
